# Evolution of nodule stiffness might predict response to local ablative therapy: A series of patients with hepatocellular carcinoma

**DOI:** 10.1371/journal.pone.0192897

**Published:** 2018-02-14

**Authors:** Michael Praktiknjo, Viktoria Krabbe, Alessandra Pohlmann, Matthias Sampels, Christian Jansen, Carsten Meyer, Christian P. Strassburg, Jonel Trebicka, Maria A. Gonzalez Carmona

**Affiliations:** 1 Department of Internal Medicine I, University of Bonn, Bonn, Germany; 2 Department of Radiology, University of Bonn, Bonn, Germany; 3 European Foundation for the Study of Chronic Liver Failure - EF CLIF, Barcelona, Spain; Medizinische Fakultat der RWTH Aachen, GERMANY

## Abstract

**Background:**

Early information on treatment response of HCC to local ablative therapy is crucial. Elastography as a non-invasive method has recently been shown to play a potential role in distinguishing between benign and malignant liver lesions. Elastography of hepatocellular carcinoma (HCC) in early response to local ablative therapy has not been studied to date.

**Methods:**

We prospectively included a cohort of 14 patients with diagnosis of HCC who were treated with local ablative therapy (transarterial chemoembolization, TACE and/or radiofrequency ablation, RFA). We used 2D shear-wave elastography (RT 2D-SWE) to examine stiffness of HCC lesion before and 3, 30 and 90 days after local ablative therapy. Contrast-enhanced imaging after 90 days was performed to evaluate treatment response. Primary endpoint was stiffness of HCC in response to local ablative therapy. Secondary end point was tumor recurrence.

**Results:**

Stiffness of HCC nodules and liver showed no significant difference prior to local ablative therapy. As early as three days after treatment, stiffness of responding HCC was significantly higher compared to non-responding. Higher stiffness before treatment was significantly associated with tumor recurrence.

**Conclusion:**

Nodule stiffness in general and RT 2D-SWE in particular could provide a useful tool for early prediction of HCC response to local ablative therapy.

## Introduction

In recent years, liver stiffness assessed by different elastography techniques (transient, shear wave, magnetic resonance, etc.) has become a standard tool for non-invasive staging of liver fibrosis and detection of clinically significant portal hypertension in patients with chronic liver disease [1–3]. Patients with chronic liver disease regularly show hepatic nodules with an increased risk of malignant transformation to hepatocellular carcinoma (HCC) [4,5]. While contrast-enhanced imaging is the first-line approach in the diagnosis of HCC, recent reports show that the non-invasively measured stiffness of hepatic nodules could help to distinguish between malignant and benign liver lesions. Interestingly, the higher the stiffness of a lesion, the higher the probability of malignancy [6–8]. The treatment of HCC depends on the stage of liver disease and in many cases, curative resection is not possible. Therefore, local ablative therapies, such as transarterial chemoembolization (TACE) and radiofrequency ablation (RFA), are considered viable therapeutic options [4]. However, HCC might behave very differently and could show more or less of a response to local ablative treatment [9]. Furthermore after treatment, recurrence of HCC, other than the target lesions (non-target lesions, Non-TL) is crucial for patient outcome and therapeutic strategy [10]. This prospective study investigated for the first time whether magnitude and development of nodule stiffness assessed by real-time two-dimensional shear wave elastography (RT 2D-SWE) is associated with response of HCC to local ablative therapy and occurrence of Non-TL.

## Materials and methods

### Patient cohort

**Fig 1:**
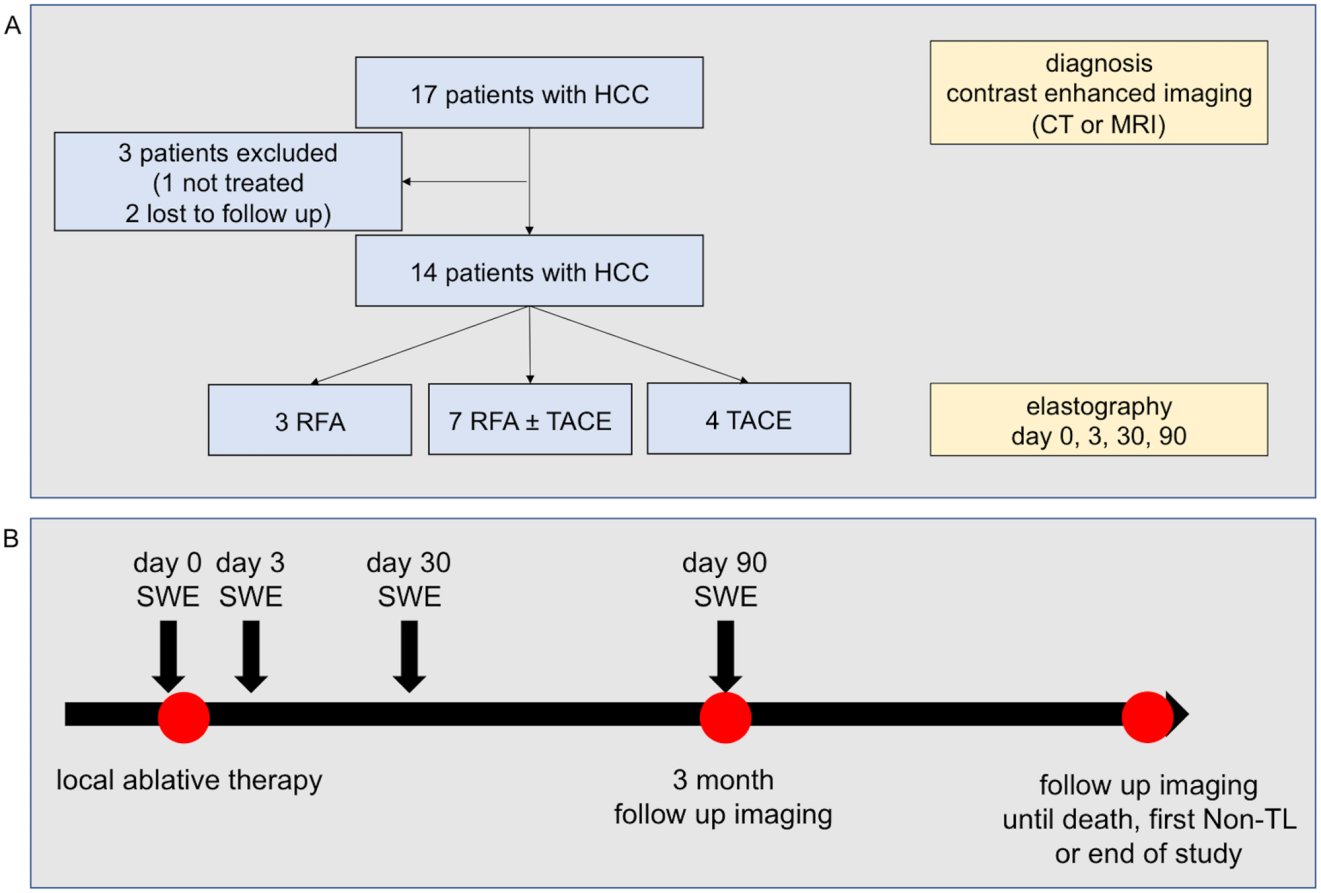
Study flow (A) and study design (B).

This study prospectively included a total of 14 patients with HCC diagnosed by typical contrast-enhanced imaging (CT and/or MRI) and were referred to local ablative therapy by our institution’s interdisciplinary tumor board. HCC that were previously treated were excluded. In total, three patients were excluded (one patient was not treated due to liver failure, two were lost to follow-up). A flow chart of patient selection is shown in Fig 1A. This study was approved by the local ethics committee (klinisches Ethikkomitee des Universitätsklinikums Bonn, number 121/14). All clinical investigations have been conducted according to the principles expressed in the declaration of Helsinki. Informed written consent has been obtained from the participants.

### Real-time two-dimensional shear wave elastography

We included only lesions that were well visible in B-mode sonography in the right liver lobe for real-time two-dimensional shear wave elastography (RT 2D-SWE) (Aixplorer, SuperSonic Imagine, France). The region of interest was set according to the size of the evaluated lesion. The assessment was performed according to manufacturer’s recommendations. RT 2D-SWE examinations were performed in a fasting condition, supine posture and intercostal probe position. At least three viable measurements were taken and mean values in kPa were recorded. In addition, RT 2D-SWE of the non-HCC liver parenchyma was assessed according to manufacturer’s recommendations and with the region of interest set at a diameter of at least 15 mm, at least 2 cm away from the HCC nodule and at least 1.5 cm deep from the liver capsule.

We examined RT 2D-SWE of HCC before local ablative therapy as well as three, 30 and 90 days after local ablative treatment. The lesion of interest was selected based on the planned treatment. Therefore, only lesions that were treated with local ablation were measured and included.

### Diagnostic work-up of HCC

The diagnosis of HCC was done with contrast-enhanced imaging (multidetector row CT or magnetic resonance imaging (MRI)). Commercially available clinical 3.0 Tesla MR imaging system (Ingenia 3.0 T; Philips Healthcare, Best, Netherlands) or a 1.5 Tesla MR imaging system (Ingenia 1.5 T; Philips Healthcare, Best, Netherlands) or CT imaging systems (Philips Brilliance 64 or Philips Brilliance 256 iCT, both Philips Healthcare, Best, the Netherlands) were used. HCC was diagnosed only if typical imaging features were detected [11]. Follow-up imaging was also performed by contrast-enhanced imaging (CT or MRI). Response to treatment was determined by contrast-enhanced imaging at three months (Fig 1b). Response to local ablative therapy was defined as lack of contrast enhancement in the lesion of interest as sign of vital tumor mass. Non-response was defined as detection of vital tumor mass. Additionally, response was also classified according to RECIST criteria [12]. Response was defined as RECIST complete remission (CR), while no response was defined as RECIST stable disease (SD) or progressive disease (PD). Follow up imaging was then performed 90 days after local ablative therapy and further recorded until the end of study, death or occurrence of Non-TL.

### Statistical analysis

Descriptive statistics were computed for all variables. Non-parametric testing was used to compare different groups when suitable. Paired non-parametric testing was used to compare data during follow-up of the same patients. Continuous variables are presented as mean ± standard error of the mean (SEM) or median (range). Categorical variables are presented as absolute cases or percentage. Distinguishing cut-off values was performed using receiver operating characteristics analysis. Time-to-Event analysis was performed using Kaplan-Meier curve with log-rank test. All data were analyzed using SPSS (version 23, IBM, Armonk, NY, USA) and plotted using Prism (version 6, GraphPad, LaJolla, CA, USA).

## Results

### Patient characteristics

A total of 14 patients with HCC were included (Fig 1A). Eight patients were male, median age was 69 years. Etiology of liver disease was viral hepatitis in seven and alcoholic liver disease in two patients. 3 patients received radiofrequency ablation (RFA), 7 RFA in combination with transarterial chemoembolization (TACE), while 4 patients received only TACE. The patients had a median of 1.5 HCC nodules with a median size of 20 mm. Median Child-Pugh and MELD scores were 6 and 12, respectively (Table 1).

**Table 1:** Patient characteristics before local ablative therapy, stratified for occurrence of Non-TL.

Parameter	all	no Non-TL	Non-TL	p
Sex (male/female)	8/6	5/1	3/5	0.181
Age	70.5(60–81)	70.5(62–81)	71(60–81)	1.000
SWE mean HCC [kPa]	18.6(8.5–39.7)	12.7(8.5–24.5)	28.2(18.0–39.7)	**0.008**
SWE mean liver [kPa]	28.2(19.1–85.0)	27.4(24.9–47.9)	31.1(19.1–85.0)	0.490
Size of largest HCC nodule [mm]	20(15–40)	22(18–49)	19.5(15–40)	0.228
Number of HCC nodules	1.5(1–4)	1(1–4)	1.5(1–4)	0.414
Type of local ablation (RFA/RFA+TACE/TACE)	3/7/4	1/3/2	2/4/2	0.852
Etiology cirrhosis (alcohol/viral/others)	5/7/2	3/3/0	2/4/2	0.282
Child-Pugh stage (A/B/C)	8/6/0	3/3/0	5/3/0	0.640
Child-Pugh score	6(5–8)	6.5(5–7)	5.5(5–8)	0.755
MELD score	12(6–22)	13.5(7–16)	9(6–22)	0.282
Serum sodium [mmol/l]	139.5(127–145)	140(138–143)	138.5(127–145)	0.491
Serum creatinin [mg/dl]	0.9(0.5–2.0)	1.0(0.5–2.0)	0.9(0.8–1.6)	0.950
Serum bilirubin [mg/dl]	1.2(0.8–3.6)	1.5(0.8–3.6)	1.2(0.9–3.3)	0.755
INR	1.2(1.0–1.9)	1.2(1.0–1.8)	1.2(1.0–1.9)	0.573
White blood cell count [G/l]	3.9(2.5–9.9)	4.3(2.5–9.9)	3.9(2.6–9.6)	0.852
Thrombocyte count [G/l]	98(29–186)	103.5(29–186)	93.5(48–165)	0.573
Serum alfa-feto protein [IU/ml]	14.1(2.2–179.3)	9.7(2.2–75.6)	22.8(3.9–179.3)	0.282

Abbreviations: Non-TL (non-target lesion), RT 2D-SWE (real-time two-dimensional shear wave elastography), HCC (hepatocellular carcinoma), TACE (transarterial chemoembolization), RFA (radiofrequency ablation), MELD (model of end-stage liver disease), mm (millimeters), kPa (kilopascals).

**Fig 2:**
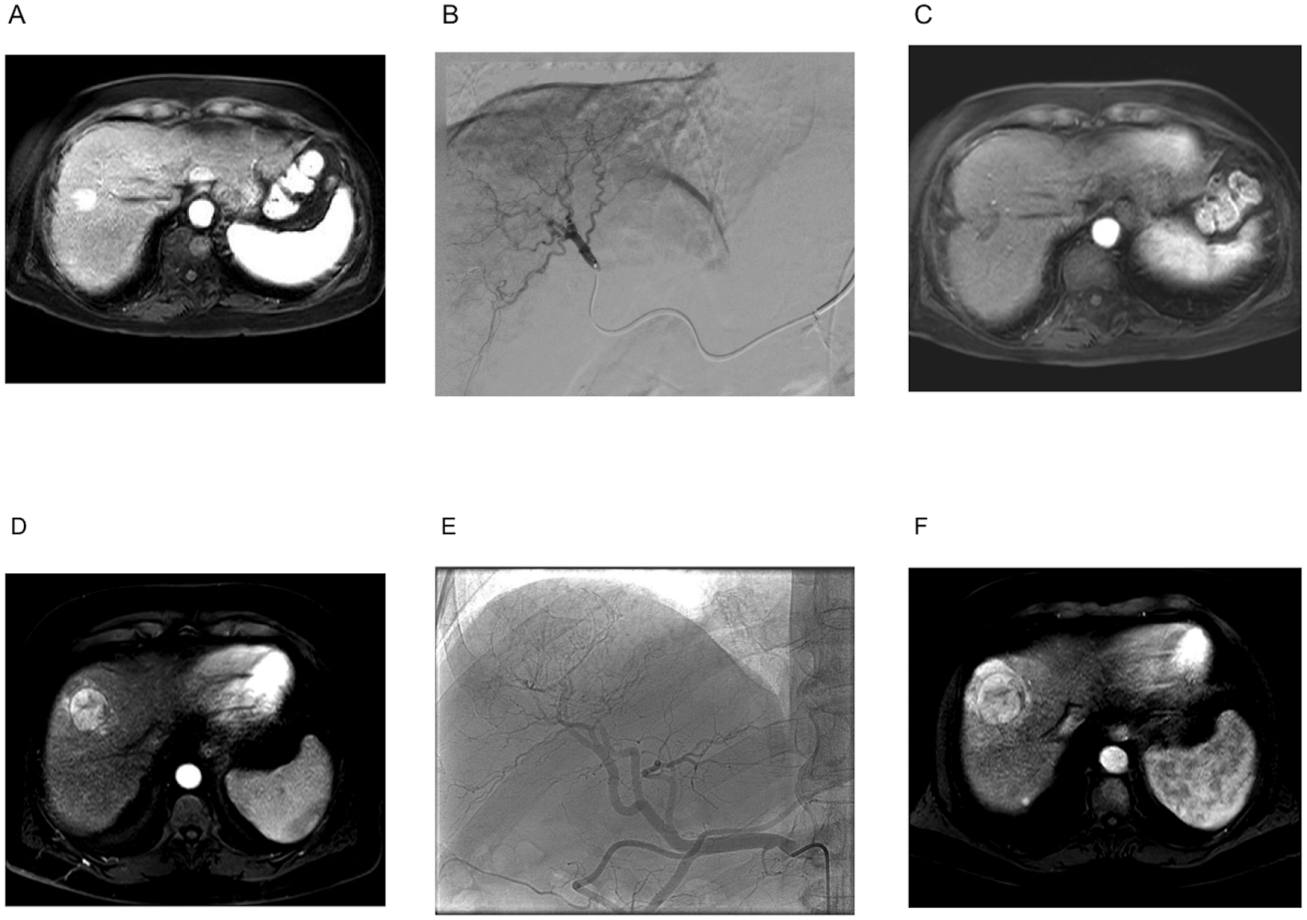
Example magnetic resonance imaging before and after local ablative therapy. A) MRI of an HCC before treatment of a responding patient. HCC shows contrast enhancement in T1 sequence (arrow). B) Angiography image taken during TACE. HCC lesion is pooling arterial flow (arrow). C) Follow-up MRI of the responder patient. The lesion only shows ablation lesion after RFA/TACE without contrast enhancement. D) MRI of an HCC before treatment of a non-responding patient. HCC shows contrast enhancement in T1 sequence (arrow). E) Angiography image taken during TACE. HCC lesion is pooling arterial flow (arrow). F) Follow-up MRI of the responder patient. The contrast-enhancing lesion shows increase in size (arrow).

After completion of the 3-months follow-up, the subsequent contrast-enhanced imaging revealed that 11 patients had complete local response to local ablative treatment (Fig 2A, 2B and 2C), while 3 patients had not complete response at the target lesion (Fig 2D, 2E and 2F). Comparison of the characteristics at baseline between target responders and non-responders showed that responders were significantly younger than non-responders, while non-responders tended towards less severe liver disease as assessed by the Child-Pugh and MELD score. All patients treated with RFA ± TACE were responders as expected. Importantly, no further baseline characteristics were significantly different, especially regarding stiffness of the liver and HCC nodules (Table 2).

**Table 2:** Patient characteristics before local ablative therapy, stratified for local response.

Parameter	all	local response	no local response	p
Sex (male/female)	8/6	5/6	3/0	0.170
Age	70.5(60–81)	69(60–81)	76(62–78)	0.769
SWE mean HCC [kPa]	18.6(8.5–39.7)	18.9(8.63–39.7)	18.0(8.5–38.7)	0.769
SWE mean liver [kPa]	28.2(19.1–85.0)	28.1(23.2–53.1)	28.3(19.1–85.0)	1.000
Size of largest HCC nodule [mm]	20(15–40)	20(15–40)	20(15–40)	1.000
Number of HCC nodules	1.5(1–4)	1(1–4)	2(2–4)	.0225
Type of local ablation (RFA/RFA+TACE/TACE)	3/7/4	3/7/1	0/0/3	**0.011**
Etiology cirrhosis (alcohol/viral/others)	5/7/2	4/5/2	1/2/0	0.885
Child-Pugh stage (A/B/C)	8/6/0	6/5	2/1	1.000
Child-Pugh score	6(5–8)	6(5–8)	5(5–7)	0.555
MELD score	12(6–22)	13(6–22)	9(8–12)	0.368
Serum sodium [mmol/l]	139.5(127–145)	140(127–145)	139(138–139)	0.456
Serum creatinin [mg/dl]	0.9(0.5–2.0)	1.0(0.5–2.0)	0.8(0.8–0.8)	0.088
Serum bilirubin [mg/dl]	1.2(0.8–3.6)	1.2(0.8–3.63)	1.2(1.2–1.9)	1.000
INR	1.2(1.0–1.9)	1.2(1.0–1.9)	1.2(1.1–1.3)	0.769
White blood cell count [G/l]	3.9(2.5–9.9)	3.7(2.5–9.9)	7.6(4.9–9.6)	0.088
Thrombocyte count [G/l]	98(29–186)	98(29–186)	98(76–165)	0.659
Serum alfa-feto protein [IU/ml]	14.1(2.2–179.3)	20.6(2.2–179.3)	4.8(3.9–13.6)	0.088

Abbreviations: Non-TL (non-target lesion), RT 2D-SWE (real-time two-dimensional shear wave elastography), HCC (hepatocellular carcinoma), TACE (transarterial chemoembolization), RFA (radiofrequency ablation), MELD (model of end-stage liver disease), mm (millimeters), kPa (kilopascals).

Of the 14 patients, 8 developed tumor recurrence (Non-TL) during follow up at a median time of 220 days (82–371). Stratifying the patients for development of Non-TL showed no significant difference in general characteristics. Of note, the stiffness of the target HCC nodule before treatment was significantly higher in the patients without Non-TL compared to those who develop Non-TL (Table 1).

Two patients died during follow up. One due to gall bladder perforation 5 months after treatment and one due to progressive tumor disease 10 months after treatment.

### Dynamics of liver stiffness

**Fig 3:**
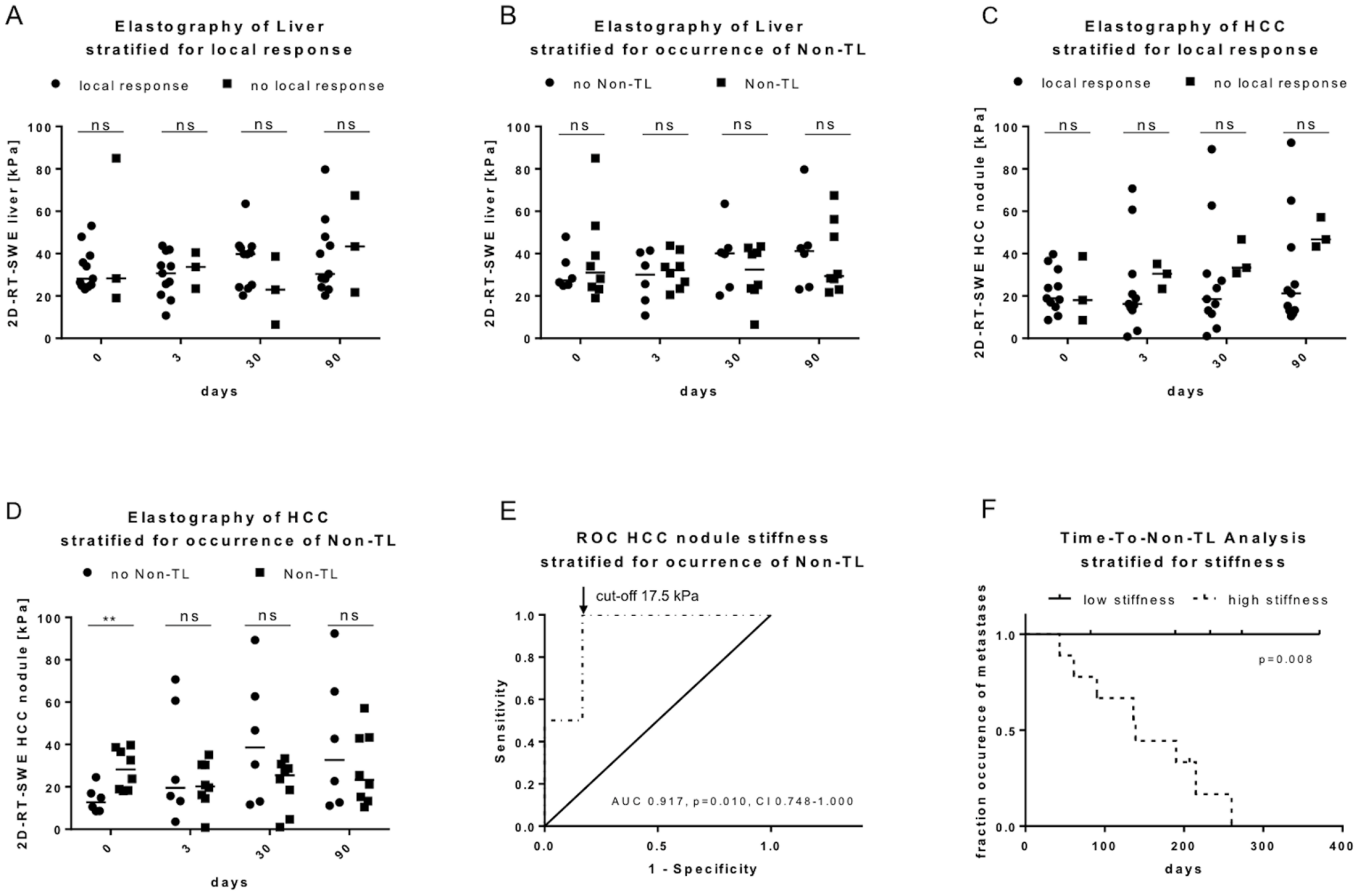
Dynamics of HCC and liver stiffness. A) Dynamic in changes in median stiffness of the liver at different time points before and after local ablative therapy stratified for local response. B) Dynamic in changes in median stiffness of the liver at different time points before and after local ablative therapy stratified for occurrence of Non-TL. C) Dynamic in changes in median stiffness of HCC at different time points before and after local ablative therapy stratified for local response. D) Dynamic in changes in median stiffness of the HCC at different time points before and after local ablative therapy stratified for occurrence of Non-TL. E) Receiver Operating Analysis for stiffness of HCC before treatment with occurrence of Non-TL as end point. Arrow shows optimal cut-off value of 17.5 kPa. F) Time-To-Event analysis with occurrence of Non-TL as endpoint stratified for HCC stiffness before treatment below and above 17.5 kPa. Abbreviations: Non-TL (non-target lesion), RT 2D-SWE (real-time two-dimensional shear wave elastography), HCC (hepatocellular carcinoma), ROC (receiver operating characteristics), kPa (kilopascals), area under the curve (AUC), 95% confidence interval (95%-CI). ns not significant, * p<0.05, **p<0.01, ***p<0.001.

Median stiffness of the non-infiltrated liver before local ablative therapy was 28.2 kPa, which is above healthy liver stiffness and in line with diagnosis of liver cirrhosis. There was no significant difference between responding and non-responding patients (Fig 3A). As expected, after local ablative therapy, at all time points, liver stiffness did not change significantly and importantly, there was no difference between responders and non-responders (Fig 3A). Stratifying the patients for occurrence of Non-TL, the results for the non-infiltrated liver stiffness are similar as shown in Fig 3b. We also analyzed the stiffness of the spleen. However, we did not find any relation with the response to local ablative therapy.

### Dynamics of stiffness of HCC in relation to local response and Non-TL development

Comparing the patients with local response to those without, the stiffness of HCC nodules was not significantly different. However, the stiffness of the nodules of the non-responding patients tended to being higher (3 days p = 0.170; 30 days p = 0.126; 90 days p = 0.126) (Fig 3C). Interestingly, when stratifying the patients for the development of Non-TL, the Non-TL group showed significantly higher stiffness of HCC before treatment (Fig 3D). After treatment the differences there were no significant differences detectable.

### Distinguishing Non-TL development with elastography

Due to this result, we aimed to identify a possible cut-off value to distinguish the patients who develop Non-TL with elastography of the HCC nodule. We therefore performed ROC analysis and found the optimal cut-off value at 17.5 kPa (sensitivity 1.000, specificity 0.833; AUC 0.917, p = 0.010, 95%-CI 0.748–1.000) (Fig 3E). We then classified the patients according to the cut-off value of 17.5 kPa into two groups: low and high stiffness. Except for one, all patients in the high stiffness group developed Non-TL, while all patients in the low stiffness group did not (Fig 3F).

### Dynamics of stiffness of HCC in relation to RECIST criteria

RECIST criteria are still commonly used in tumor imaging and therefore we also rated the patients according to RECIST. As shown in the general characteristics in Table 3 patients with no complete response RECIST response had significantly more often only TACE and tended to have more HCC nodules. All patients with complete RECIST response received RFA ± TACE, while all TACE patients were non-responders (Table 3).

**Table 3:** Patient characteristics before local ablative therapy, stratified for RECIST response.

Parameter	all	RECIST CR	RECIST SD+PD	p
Sex (male/female)	8/6	3/5	5/1	0.181
Age	70.5(60–81)	67(60–81)	75(63–76)	0.108
SWE mean HCC [kPa]	18.6(8.5–39.7)	21.0(8.6–39.7)	18.5(8.5–38.7)	0.852
SWE mean liver [kPa]	28.2(19.1–85.0)	27.3(23.2–53.1)	31.2(19.1–85.0)	0.755
Size of largest HCC nodule [mm]	20(15–40)	19.5(15–30)	22.5(15–40)	0.228
Number of HCC nodules	1.5(1–4)	1(1–4)	2(1–4)	0.081
Type of local ablation (RFA/RFA+TACE/TACE)	3/7/4	3/5/0	0/2/4	0.043
Etiology cirrhosis (alcohol/viral/others)	5/7/2	3/4/1	2/3/1	0.852
Child-Pugh stage (A/B/C)	8/6/0	4/4/0	4/2/0	0.533
Child-Pugh score	6(5–8)	6.5(5–8)	5(5–7)	0.282
MELD score	12(6–22)	13.5(8–22)	8.5(6–15)	0.081
Serum sodium [mmol/l]	139.5(127–145)	139(127–143)	139.5(138–145)	0.573
Serum creatinin [mg/dl]	0.9(0.5–2.0)	1.0(0.5–1.6)	0.9(0.8–2.0)	0.662
Serum bilirubin [mg/dl]	1.2(0.8–3.6)	2.5(0.9–3.6)	1.1(0.8–1.9)	0.059
INR	1.2(1.0–1.9)	1.3(1.1–1.9)	1.1(1.0–1.3)	**0.043**
White blood cell count [G/l]	3.9(2.5–9.9)	3.3(2.5–5.7)	6.5(3.8–9.9)	**0.008**
Thrombocyte count [G/l]	98(29–186)	72(29–109)	116.5(76–186)	**0.043**
Serum alfa-feto protein [IU/ml]	14.1(2.2–179.3)	22.7(5.4–179.3)	9.2(2.2–50.0)	0.081

Abbreviations: Non-TL (non-target lesion), RT 2D-SWE (real-time two-dimensional shear wave elastography), HCC (hepatocellular carcinoma), TACE (transarterial chemoembolization), RFA (radiofrequency ablation), MELD (model of end-stage liver disease), mm (millimeters), kPa (kilopascals).

**Fig 4:**
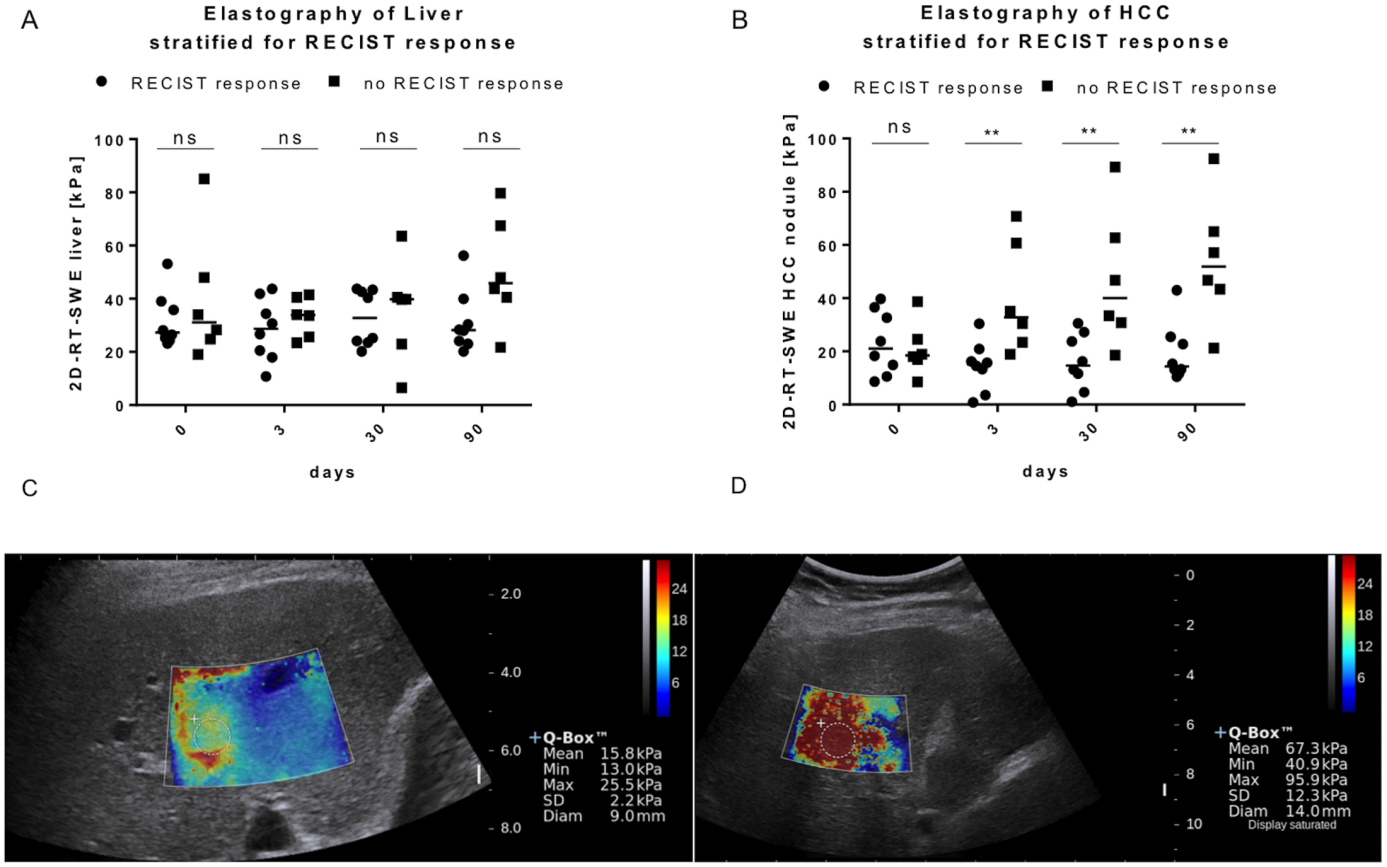
Dynamics of HCC and liver stiffness and examples. A) Dynamic in changes in median stiffness of the liver at different time points before and after local ablative therapy stratified for local response. B) Dynamic in changes in median stiffness of the liver at different time points before and after local ablative therapy stratified for occurrence of Non-TL. Example of an HCC nodule with low C) and high D) stiffness after local ablative therapy.

As early as three days after treatment, nodule stiffness of the RECIST responders remained stable, while HCC stiffness of RECIST non-responders increased, rendering the difference between response and non-response statistically significant (Fig 4B). Similarly, one and three months after local ablative treatment, HCC RECIST non-responders showed a significantly higher stiffness of the lesion compared to responders (Fig 4B, 4C and 4D). As expected non-infiltrated liver stiffness did not change significantly (Fig 4A). Therefore, the nodule-to-liver-stiffness ratio revealed no statistically significant differences between responders and non-responders.

Probably due to the small sample size, none of the assessed parameters were significantly and independently associated with non-response, including, in particular, treatment modality, evolution of elastography, and severity of disease.

## Discussion

This study provides first data of non-invasively measured HCC stiffness in response and outcome to local ablative therapy. We not only demonstrate that stiffness before treatment might distinguish patients at risk of developing Non-TL but also show that an increase in stiffness as early as three days after therapy could be useful in predicting outcome of local ablative therapy according to RECIST criteria.

Local recurrence and residual tumor mass in local ablative therapy is more frequent compared to surgical resection [13]. Therefore, accurate information on tumor response is crucial for patient outcome and future therapeutic strategies, such as repeated local ablation [14]. However, in most institutions, follow-up contrast-enhanced imaging to evaluate successful treatment is usually performed not before one to three months after treatment [15,16]. Efforts for an earlier assessment of successful treatment response have been made but would involve special CT protocols using specific software [17].

Elastography has been shown to be useful in distinguishing between benign and malignant focal liver lesions [6,8,18,19]. Whilst classification of malignant liver lesions might be accurate with RT 2D-SWE, almost 50% of lesions cannot be classified due to high variability in stiffness, which is the most significant elastographic feature of HCC [8,20]. Our patients with higher HCC stiffness were at high risk to develop Non-TL after local ablation. Our study also shows that measurement of HCC stiffness soon after local ablative therapy could predict response to local ablative therapy. These results seems feasible as biologically HCC are very diverse and differences in aggressive behavior of HCC might affect treatment response [9]. The significant difference in HCC stiffness possibly reflects these different biological entities of HCC [21].

It has been shown that some HCC have microvascular invasion and/or micrometastases [22,23]. Depending on the aggressiveness of tumor biology complex vascular anatomy and hyperperfusion in these tumors might be more prominent [21,24]. Stiffness, upon other factors, is dependent on vascularity, blood flow and fibrous tissue and therefore might cause different levels of stiffness in different subsets of HCC. For the liver in general it has been shown that an arterialization of blood perfusion leads to an increased non-invasively measured stiffness [25]. With this knowledge in mind, it seems feasible that more aggressive HCC with stronger neo-vascularization (in case of HCC mainly arterial) might appear with higher values of stiffness.

One might argue that after RFA mostly the treated HCC nodules are completely ablated and the lesion after treatment therefore only consists of necrosis [26]. Nevertheless, while local tumor control of the target lesion is important, the recurrence of HCC in Non-TL is also crucial for the patients’ outcome and future therapeutic strategies. Therefore, the ability to predict those outcomes without biopsy is needed and our data suggest a role of elastography after treatment in this setting.

Importantly, since—according to the MELD score—non-responders had an even better liver function than responders, it is unlikely that HCC stiffness was due to an overall increased liver stiffness in these patients (which was similar). Rather, this was due to the mechanical and biological properties of the nodules.

This work has several limitations, mainly the small sample size and therefore serves more as a proof-of concept study. Another limitation of the study might be a variability in measurements depending on a different of factors like volume status of the patients. Of note, in our study, the HCC-to-liver stiffness ratio did not provide significant predictive value, which is in line with previous studies [8] and underlines the robustness of our results, despite the small sample size. Furthermore, to confirm the hypothesis that different biological entities of HCC are represented by different types of stiffness of the nodules, further studies with more patients and considerably more elaborate histologic work-up are required. However, this study presents a real-life clinical setting, where diagnosis is often based only upon contrast-enhanced imaging and where biopsy is not recommended to be performed regularly [4,5].

Non-invasive evaluation of stiffness of HCC nodules before and after local ablative treatment is feasible and an easy and promising tool to predict response long before the usual contrast enhanced imaging.

## Abbreviations

HCC: hepatocellular carcinoma
MELD: model for end-stage liver disease
RFA: radiofrequency ablation
RT 2D-SWE: real-time two-dimensional shear wave elastography
TACE: transarterial chemoembolization
